# Nuclear localization of B7-H4 in pulmonary adenocarcinomas presenting as a solitary pulmonary nodule

**DOI:** 10.18632/oncotarget.10542

**Published:** 2016-07-12

**Authors:** Cheng Chen, Wei-Dong Zhu, Fang Xie, Jian-An Huang

**Affiliations:** ^1^ Respiratory Department, The First Affiliated Hospital of Soochow University, Suzhou, 215006, China; ^2^ Pathology Department, The First Affiliated Hospital of Soochow University, Suzhou, 215006, China; ^3^ Pathology Department, Soochow University, Suzhou, 215006, China

**Keywords:** B7-H4, solitary pulmonary nodule, adenocarcinoma, ground-glass opacity

## Abstract

**Purpose:**

Although the pathogenicity of B7-H4 in cancer is well established, its role in pulmonary adenocarcinoma, especially lesions presenting as solitary pulmonary nodules (SPNs), remains unclear.

**Methods:**

40 cases of pulmonary adenocarcinoma presenting with SPN were enrolled during year 2012–2015. The B7-H4 expression and its subcellular distribution in pulmonary adenocarcinoma presenting with SPN were analyzed by immunohistochemistry, further its correlation with Ki-67 expression and CT feature. *In vitro*, the B7-H4 expression in the cytoplasmic and nucleus fractions of lung cancer cell lines was determinate by western blotting.

**Results:**

Immunostaining revealed B7-H4 in the cytoplasm of cells from all 40 SPN samples studied. No surface localization of B7-H4 was detected, but in 18 samples the nuclear membranes were B7-H4-positive. Moreover, patients with more poorly differentiated and invasive adenocarcinomas showed greater localization of B7-H4 to the nuclear membrane. The percentage of lesions with ground-glass opacity was significantly greater among samples negative for nuclear membrane B7-H4. Most importantly, there was a statistically significant relationships between the Ki-67 index and B7-H4 positivity of the nuclear membrane. This suggests tumors exhibiting higher nuclear membrane B7-H4 have greater proliferative potential. Western blotting confirmed both cytoplasmic and nuclear B7-H4 localization in lung adenocarcinoma cell lines.

**Conclusions:**

Taken together, our study provides a new insight into the tumorigenicity of B7-H4 in lung adenocarcinoma. We suggest that in pulmonary adenocarcinoma presenting with SPN, nuclear membrane localization of B7-H4 within the tumor cells is associated with increased malignancy.

## INTRODUCTION

Solitary pulmonary nodules (SPNs) are single lesions in the lung that are ≤ 3 cm in diameter, without obstructive pneumonia or atelectasis involving lung segments or lobes. Although some SPNs are detected pathologically during the early stages of lung cancers, most are not malignant. For that reason, attention is being devoted to the biological behavior of SPNs and to whether they are predictive of aggressiveness. In part to address recent advances in our understanding lung adenocarcinoma presenting as SPNs, new classifications were purposed by the relevant international multidisciplinary committees [1–2]. According to those classifications, adenocarcinomas are classified as acinar, papillary, micropapillary, lepidic and solid.

Among SPNs, ground glass nodules (GGNs) were frequently seen on computed tomography (CT). As the size and density of these malignant GGNs increased, their lepidic component gradually decreased, and were replaced by acinar or papillary components [3–4]. This type of change in the composition of tumor cells is thought to be a specific feature of carcinogenesis [5]. For example, the ground-glass opacity (GGO) ratio in SPN is strongly associated with survival, and GGO-dominant tumors have a 5-year survival rate of nearly 100% [6]. To date, however, the molecular changes in tumor composition during SPN growth has not been fully characterized.

B7 is a ligand membrane protein that provides a costimulation signal in T cells. B7-H4 is a B7 ligand that exerts a immunosuppressive effect in diseases such as cancer, allograft rejection and autoimmune diseases [7–10]. B7-H4 overexpression is seen in various types of human tumors, including renal cell carcinoma, ovarian cancer, gastric cancer, breast cancer and lung cancer, where it plays an important part in tumor progression and is associated with a poor prognosis [11–15]. In addition, Yao et al. recently demonstrated a direct relationship between World Health Organization (WHO) grading of gliomas and the levels of B7-H4, suggesting B7-H4 could serve as a potential marker of glioma carcinogenesis [16]. But although the tumorigenicity of B7-H4 has been well established, few studies have focused on its function in pulmonary adenocarcinoma presenting as SPNs.

## MATERIALS AND METHODS

### Study subjects

The study subjects comprised 40 patients (10 male and 30 female) with SPNs who underwent thoracotomy or selected video-assisted thoracic surgery (VATS) between 2012 and 2015. The study was approved by the ethics committee of The First Affiliated Hospital of Soochow University.

### Cell lines and mAbs

The H1299, A549, SPCA-1, H1650 and H1975 cell lines were purchased from the Chinese Academy of Sciences (Shanghai, China). The anti-Ki-67 (Genetech, clone GM001), anti-B7-H4 (EP1165, Novus Biologicals), anti-PARP (Beyotime China) and anti-a-tubulin (Beyotime China) mAbs were used.

### CT image acquisition

All image examinations were conducted using a CT scanner (Philips Brilliance 16, kV: 120.00 or Siemens, kV: 100.00). CT scans were used to assess tumor size and density in the lung setting. The images were displayed at a window width of 1000 Hounsfield units (HU) and a level of −750 HU within the lung window and at a window width of 400 HU and a level of 45 HU within the mediastinal window.

### Pathology examination

Archived paraffin blocks were cut into 4-μm-thick sections and transferred to polylysine glass slides. All slides were classified based histological type and tumor grade according to WHO guidelines.

### Immunohistochemistry

Formalin-fixed and paraffin-embedded sections were reviewed with selection of representative sections for immunohistochemistry. Briefly, 4- μm-thick sections were mounted on Superfrost microscopic slides, deparaffinized and dehydrated. Antigen retrieval was accomplished by pressure cooking. The slides were incubated with anti-Ki-67 or anti-B7-H4 mAb [17–18].

### Evaluation of staining

Ki-67 immunopositivity was defined as the percentage of immunoreactive tumor cells among the total number of tumor cells (Leica Microsystems). Evaluation of the B7-H4 staining was based on subcellular positivity of tumor cells. The presence of B7-H4 expression in stromal cells from samples could be excluded by virtue of their histomorphological features and topographic location within tissue sections.

### Western blotting

Cells were washed with PBS and dispersed using 0.2% EDTA. The cytoplasmic and nuclear protein fractions were prepared using a Nuclear and Cytoplasmic Protein Extraction Kit (Beyotime Institute Biotechnology, Nantong, China) according to the manufacturer's instructions. The cellular fractionation efficiency was assessed using antibodies against tubulin and PARP. Proteins were separated using 12% SDS-polyacrylamide gel electrophoresis. Following equilibration for 2 h in transfer buffer (20% methanol, 190 mM glycine, 25 mM Tris), the proteins were blotted onto polyvinylidene difluoride membranes (Millipore), which were blocked for 2 h in 5% BSA/PBS at room temperature, washed with PBS containing 0.1% Tween-20 (PBST), and incubated overnight at 4°C with a primary antibody (anti-B7-H4 mAb, anti-PARP mAb or anti-tubulin mAb) in 5% BSA/PBS. The blots were then washed and labeled for 1 h with HRP-conjugated secondary antibody.

### Statistical analysis

Statistical analysis was performed using SPSS statistical software. The Chi-squared test and 2-sided Fisher exact test were applied to determine the strength of associations. All tests were two-sided; values of *P* < 0.05 were considered significant.

## RESULTS

### Baseline characteristics of patients and pathologic diagnoses

The baseline characteristics of the 40 patients (10 men and 30 women) included in the study are listed in Table 1. The median age of the patients was 62 years (range, 37–75 years). At the time of the CT scan, the median lesion size was 1.8 cm (range, 0.6–3.0 cm), and 18 of the 40 lesions were pure/mixed GGN. All cancers were diagnosed pathologically and included 4 atypical adenomatous hyperplasias (AAH), 6 adenocarcinomas *in situ* (AIS), 8 lepidic adenocarcinomas, 4 well or moderately differentiated adenocarcinomas and 18 poorly differentiated adenocarcinomas.

**Table 1:** Patients' clinical characteristics

Factor	Number
Age Mean (range)	62 (37–75)
Gender (*n*)	
Male	10
Female	30
Differentiation	
AAH	4
AIS	6
Lepidic	8
Well	4
Poor	18
Invasiveness	
Pre-invasive	10
Invasive	30
CT image	
p/mGGO	18
Solid mass	22

Abbreviations: p/mGGO (pure/mix GGO).

### Subcellular distribution of B7-H4

**Figure 1:**
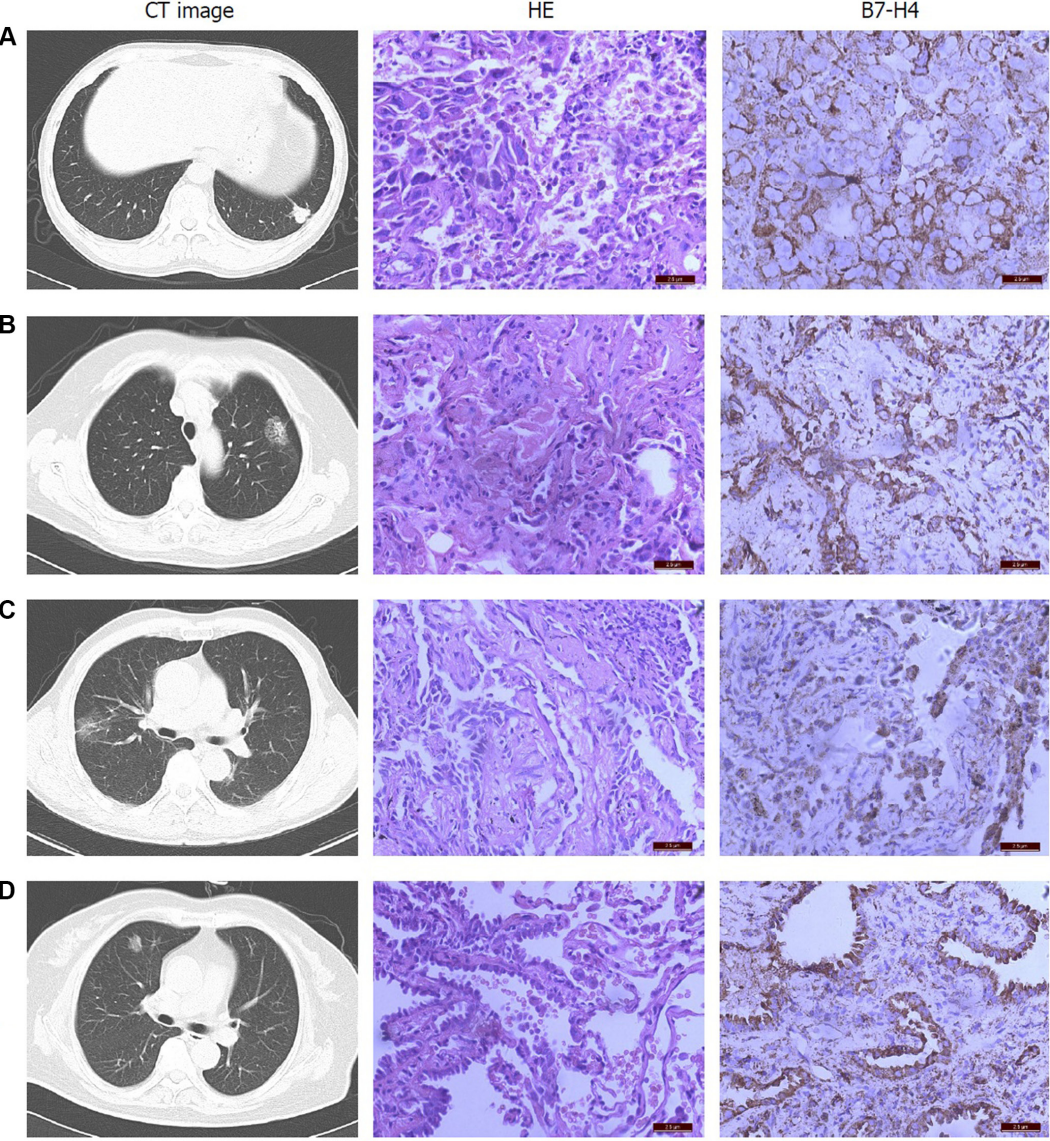
Expression of B7-H4 in pulmonary adenocarcinomas presenting as SPNs was analyzed immunohistochemically Poorly differentiated adenocarcinomas exhibiting a solid mass on CT images (**A**) were positive of B7-H4 localized at the nuclear membrane. Lepidic adenocarcinomas (**B**), adenocarcinomas *in situ* (**C**) and well-differentiated adenocarcinomas (**D**), which all exhibited GGN on CT images, did not harbor B7-H4 at the nuclear membrane.

As shown in Figure 1, *in situ* tumor cells were heterogeneous with respect to B7-H4 expression. B7-H4 was detected in the cytoplasm of all 40 samples, but no cell membranes were B7-H4-postive. Intranuclear B7-H4 was detected in 8 cases and, notably, 18 tumors showed B7-H4 positivity at the nuclear membrane.

### Pathologic features and B7-H4 expression

Evaluation of the association between pathologic parameters and nuclear expression of B7-H4 is summarized in Table 2. It appears that patients with poorly differentiated and invasive adenocarcinomas had greater localization of B7-H4 at the nuclear membrane. Univariate analysis revealed that B7-H4 localization at the nuclear membrane is significantly associated with tumor differentiation and invasiveness (*P* < 0.05).

**Table 2:** The relationship between the clinic parameters of SPN and B7-H4 expression

B7-H4 (nuclear-mem)	CT image		Invasiveness		Differentiation	
	GGN	Non-GGN	Pre-invasive	Invasive	Poor	Non-poor
Negative	18	4^*^	10	12^**^	6	18^***^
Positive	0	18	0	18	12	4

Note:

*, **, *** *P* < 0.05.

### CT features and B7-H4 expression

Based on the CT features, SPNs were classified as GGN or non-GGN. None of the 18 GGN SPNs showed localization of B7-H4 at the nuclear membrane, whereas 18 of the 22 non-GGN lesions were positive for B7-H4 at the nuclear membrane (Table 2). This suggests that greater lesion density in malignant SPNs was associated with localization of B7-H4 at the nuclear membrane within the tumor cells.

### Ki-67 expression and B7-H4 expression

Ki-67 expression is known to correlate with both cancer cell proliferation and differentiation. We were able to evaluate Ki-67 levels in 30 of the 36 evaluable primary lung cancers. The median Ki-67 index was 15%, and there was a significant association between the Ki-67 index and B7-H4 localization at the nuclear membrane (*P* < 0.05). This suggests that cancer cells with higher B7-H4 levels at the nuclear membrane have greater proliferative potential (Table 3).

**Table 3:** The relationship between the Ki-67 index and B7-H4 expression

B7-H4 (nuclear-mem)	Ki-67	
	≤15%	> 15%
Negative	14	4^*^
Positive	8	10

Note:

* *P* < 0.05.

### Subcellular distribution of B7-H4 in lung cancer cell lines

After nuclear and cytoplasmic fractionation of several lung cancer cell lines was assessed using anti-tubulin and anti-PARP mAbs, western blotting showed that B7-H4 was distributed in both the nuclear and cytoplasmic fractions (Supplementary Figure S1).

## DISCUSSION

Adenocarcinoma is the most commonly occurring type of lung cancer. The majority of primary lung adenocarcinomas have the radiologic appearance of a GGN at diagnosis, and early detection and treatment of SPNs should improve the prognosis of these patients. However, there have been no studies of the aberrant protein expression in the micropapillary component and its influence on malignancy.

B7-H4 was first identified in 2003 and was found to suppress immune response upon binding B7 on T cells. In ovarian tumor cells, B7-H4 reportedly localizes predominantly in the intracellular compartments of the cells. In contrast to membrane-bound B7-H4, the intracellular protein does not suppress T cell immune responses. Instead, it mediates cell proliferation, migration and invasion, suggesting the function of intracellular B7-H4 distinctly differs from the membrane-bound form [19–20]. Consistent with that idea, B7-H4 facilitates cell proliferation by promoting a positive IL-6/STAT3 loopback pathway in esophageal squamous cell carcinoma [21]. Additionally, Zhang et al. showed that B7-H4 is present at the nuclear membrane and in the cytosol and nucleus of renal carcinoma cells, and that it is translocated between the cytoplasm and nucleus to play direct role in mediating proliferation and cell cycle progression [17].

When evaluating a SPN diagnosed as lung carcinoma, its malignant potential and the molecular mediators present should be considered. In the present study, we identified B7-H4 as a protein that is likely translocated between the cytosol and nucleus. In addition, whereas cytosolic localization of B7-H4 was a common feature of all the cancers tested, the presence of B7-H4 at the nuclear membrane correlated significantly with greater malignancy and invasiveness. We also noted that the percentage of lesions that presented as GGO was significantly greater among tumors that were negative for B7-H4 at the nuclear membrane. Patients with poorly differentiated and invasive adenocarcinomas with greater proliferative potential (indicated by Ki-67 analysis) were more likely to exhibit high levels of B7-H4 at the nuclear membrane. Furthermore, B7-H4 expression in several lung cancer cell lines confirmed the inherent cytoplasmic and nuclear localization of B7-H4. Although very little is known about the effect of nuclear membrane B7-H4 on tumorigenesis, we suggest that translocation of B7-H4 across the nuclear membrane represents a carcinogenic event in lung adenocarcinoma as the tumor grows from GGO to a solid lesion.

A limitation of this study is that the patient sample was relatively small. Additional investigations with larger numbers of patients will be required to assess the malignant behavior of GGO-dominant tumors, especially their histological features, to accurately determine prognosis. This includes the effect of B7-H4 on disease recurrence and mortality.

In summary, this study sheds light on the molecular characteristics and histologic variation among pulmonary adenocarcinomas. Our results suggest B7-H4 is not only an immunomodulator, it is also a carcinogenic mediator in lung adenocarcinoma. If the actions of B7-H4 in the nucleus are confirmed *in vivo* and the changes in the ratio of GGO are revealed, this may enable new treatment strategies for adenocarcinoma to be developed.

## SUPPLEMENTARY MATERIALS


