# Supplementary material for: Nuclear localization of B7-H4 in pulmonary adenocarcinomas presenting as a solitary pulmonary nodule
Source: Oncotarget. 2016 Jul 12;7(36):58563–8. doi: 10.18632/oncotarget.10542 (PMC5295452; doi:10.18632/oncotarget.10542)
Supplement: Supplementary file 1 [file oncotarget-07-58563-s001.pdf]

Nuclear localization of B7-H4 in pulmonary adenocarcinomas presenting as a solitary pulmonary nodule

Supplementary Materials

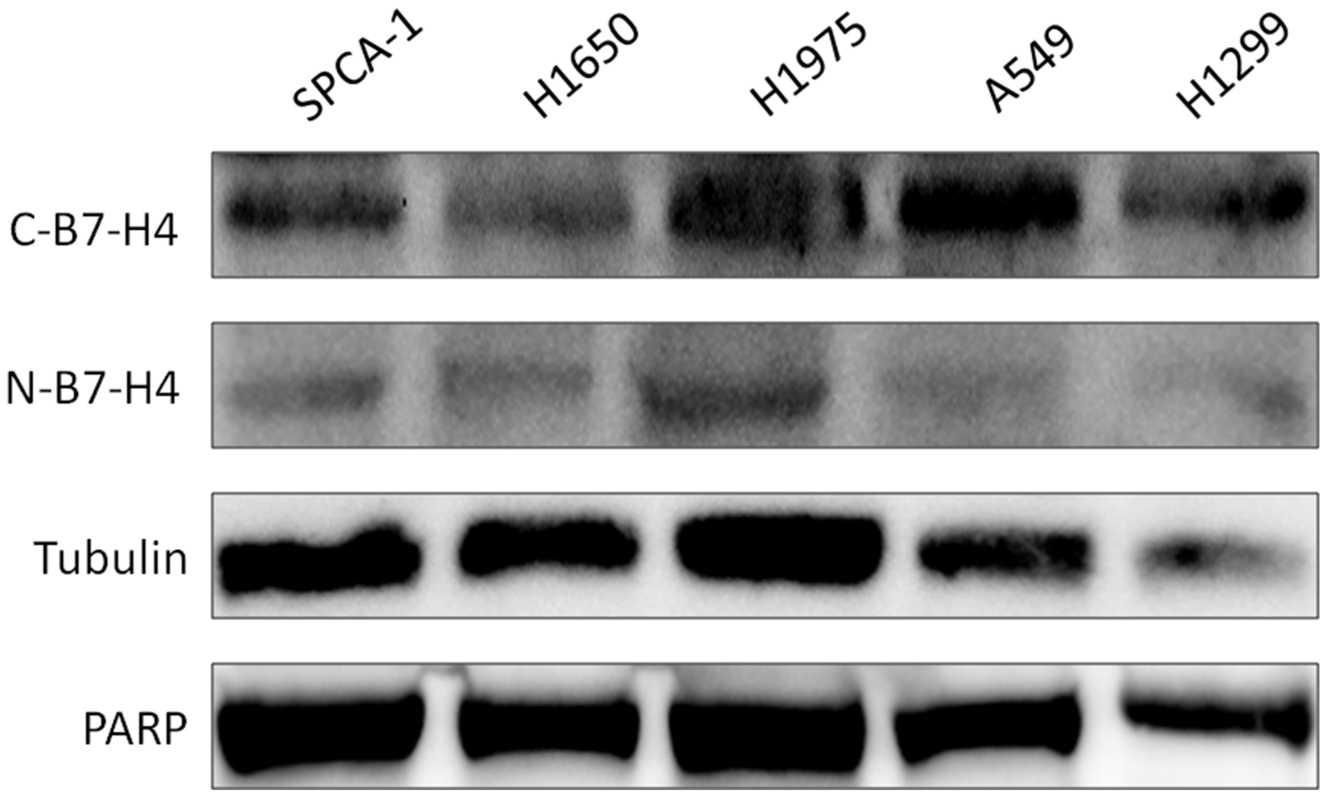

Supplementary Figure S1: Western blot analysis of expression of B7-H4 in lung cancer cell lines. The proteins from the cytoplasmic (C) and nuclear (N) fractions were blotted with anti-B7-H4. Tubulin and PARP are housekeeping proteins serving as loading controls.
